# Measurement and Decomposition of the Health Poverty of Rural Residents in China

**DOI:** 10.3390/ijerph191912876

**Published:** 2022-10-08

**Authors:** Haiyan Jia, Xiaoyu Sai, Yangyue Su, Ying Huang

**Affiliations:** 1 School of Public Administration and Policy, Shandong University of Finance and Economics, Jinan 250014, China; 2 School of Management Engineering, Shandong Jianzhu University, Jinan 250014, China

**Keywords:** rural, health poverty, health literacy, health environment

## Abstract

Narrowing the health gap and promoting health equality is the key to effectively blocking the intergenerational transmission of rural poverty. Previous studies have mainly focused on the relationship between health and poverty, but assessments of health poverty are lacking, especially with regard to the health poverty of rural residents. Based on China’s large sample household survey data, this study uses the Alkire–Foster (AF) method to measure and decompose the health poverty of rural residents. The results show that the health poverty of Chinese rural residents greatly improved from 2016 to 2018. However, significant regional differences exist with regard to the level of health poverty. The marginal contribution of economic poverty alleviation is diminishing; the equalization of health services and security has shifted to a policy focus. Community environmental management has also become an important aspect of health poverty governance, and individual health literacy and behavior have played an important role in endogenous poverty alleviation. Ultimately, this paper offers some insightful policy implications. This study extends the multidimensional poverty measurement system and reveals the relationship between health poverty and regional economic and social development. The findings also enhance the understanding of the health poverty of rural residents in developing countries.

## 1. Introduction

Ever since the “Human Development Report 2010” was released by the United Nations Development Program (UNDP), the idea of multidimensional poverty has been widely accepted globally. According to the sustainable development goals proposed by the United Nations, all forms of poverty should be eliminated worldwide by 2030 [1]. This implies that poverty governance in most low- and middle-income countries has undergone a certain transformation, and the world has entered a period of multidimensional poverty.

The existence of health problems represents an important burden for individuals in terms of deprivation. For a certain socio-economic level, material well-being is lower for people with limitations [2]. Therefore, health poverty is one of the important dimensions of multidimensional poverty measurement. Health poverty refers to the poverty caused by impaired health, or to the diseases, caused by poverty, that exacerbate existing poverty [3]. Health poverty is mainly manifested in the lack of opportunities for residents to seek medical and health care. Another manifestation is the weakening and deprivation of their ability to participate in social and economic activities, due to declining levels of health [4,5,6]. From the perspective of intergenerational transmission, this weakening and deprivation of residents’ viability also leads to reduced investment in these residents’ children’s education. Inadequate accumulation of human capital in the next generation has the potential to push individuals or families into chronic poverty [7]. Currently, 100 million people worldwide are living in poverty due to disease [8]. Solving the issues of poverty caused by disease, and a return to poverty due to disease, are major challenges in the fight against poverty. Health poverty governance is the theme of people’s livelihoods and is an issue of wide concern for all countries in the world [9,10].

Judging from existing literature, most previous studies have mainly regarded health as a form of human capital. Previous studies have focused on the relationship between health shocks, personal or family medical expenditure and poverty [11], as well as on the poverty alleviation effect of health insurance, disease treatment, etc. [12,13]. The measurement indicators of health poverty used in these studies were also relatively simple—mostly physiological indicators, such as disease risk, mortality, and life expectancy [14,15]. Entering the era of multi-dimensional poverty governance, the goal of health poverty alleviation policies in most countries has been extended to the pursuit of high-level policy areas. This includes the pursuit of equality of individual health opportunities and rights, and the equalization of health services and security. The indicators used to measure health poverty have now begun to include factors, such as the endogenous poverty alleviation motivation and self-development ability of poor individuals. Other indicators include these individuals’ interaction with the external living environment, and even the promotion of people’s health and well-being [16,17,18,19]. However, up to now, few studies have developed multidimensional measurement indicators of health poverty; empirical research on the health poverty assessment of rural residents has also not been fully carried out.

In 2020, the Chinese government announced that it had won the battle against poverty. The country’s poverty governance had achieved remarkable results; the health gap between urban and rural residents had continued to narrow, and the health poverty situation had been significantly improved [20]. However, a considerable number of rural low-income groups were still suffering from low health literacy, unequal health rights, and inadequate health protection [21]. Under the joint influence of economic and social development, the external environment, COVID-19 and other factors, the health poverty risk of rural residents has become more uncertain. The experience accumulated and the new challenges faced by China’s Health Poverty Alleviation Program (HPAP), all of which are very representative, have provided empirical evidence and future warnings for poverty governance in developing countries.

This study attempts to consider economic poverty and multidimensional health deprivation and constructs a multidimensional health poverty index (MHPI). The Alkire-Foster (AF) method is used to measure the overall change in the health poverty of rural residents in China. Subsequently, this study decomposes the health poverty index (HPI). We decompose by regions to observe the relationship between regional, economic and social development levels and the occurrence of rural health poverty. These are decomposed by indicators to observe the contribution of each indicator to rural residents’ health poverty, and to explore the micro-causes of health poverty.

This study makes the following new contributions: First, this study constructs the MHPI from the dual perspectives of the supply side and the demand side, and resets the critical value of the indicator according to the connotation of multidimensional poverty. The indicator system is more suitable for the observation of rural residents’ health poverty, and as such, this study enriches the existing research on poverty governance. Second, this study uses China’s large sample household survey data to measure and decompose the deprivation of health poverty in China’s rural areas. The causes of poverty are explored, policy goals are made clear, and solutions are proposed. This study could help policymakers formulate better public health policies, especially in developing countries, where poverty governance is moving toward multidimensional poverty.

## 2. Literature Review

Completely eliminating poverty is an eternal topic for the healthy development of human society. On the one hand, the health problems of the poor in developing countries have become increasingly prominent, and a poorer health status has aggravated the poverty level. On the other hand, health equality is an important embodiment of social justice [22]. Carrying out HPAPs can help narrow the health gap and improve the health and well-being of all mankind. By combing through the relevant literature, this study found that previous research on health and poverty has mainly focused on the aspects described below.

### 2.1. The Relationship between Poverty and Health

Research shows that health has a positive impact on income. For example, the health benefits brought by pension insurance are mainly reflected in disease prevention [23], which is an important safeguard against falling into poverty [24]. At the same time, an improvement of health status is also a necessary condition for poverty reduction [25,26]. A better level of health can reduce medical expenditure, empower human capital, and thus increase income and effectively eliminate economic poverty [27]. Aregbeshola and Khan [28] found that out-of-pocket medical expenditure would lead to a 0.8% increase in the poverty rate in Nigeria. Kim’s analysis [29] revealed that health shocks led to an increase of 4.6 to 8.0 percentage points in the proportion of absolutely poor households in South Korea. In addition, poverty is often accompanied by lower medical accessibility and affordability. Poverty therefore hinders individuals from obtaining adequate and high-quality modern medical services and thus has a negative impact on health [30,31]. Harris et al. [32] argued that poor households are more vulnerable to disease shocks and have a shorter life expectancy than non-poor households [33]. Low income, high medical expenses and a lack of medical insurance are the basic logical mechanisms of the cycle between poverty and disease [34]. 

### 2.2. The Measurement of Health Poverty

The global Multidimensional Poverty Index (MPI) was jointly released by the UNDP and the Oxford Poverty and Human Development Initiative. The MPI can be used to measure the incidence, depth and composition of multidimensional poverty, and is widely used [35,36,37]. These indices have also been developed and expanded into the area of health poverty. For example, Yan et al. [38] used mortality, cognitive health, mental health, physical health, and self-reported health (SRH) status to construct a health inequality index. Iqbal and Nawaz [39] selected the use of health services, the quality of health services, the cost of health services, and maternal and child health to generate a HPI to measure the health poverty population in rural Pakistan. Traoré [40] used indicators, such as life expectancy at birth and per capita GDP (purchasing power parity), to calculate health poverty in sub-Saharan African countries. These indices are also frequently used to assess the effects of health on poverty reduction. Some studies have found that China’s HPAP can reduce the average out-of-pocket medical expenditure of rural households by 15% [41] and can reduce the incidence of catastrophic health expenditure by 17.1% [42].

## 3. Methodology

### 3.1. Research Method

Based on the “capability approach” theory, Alkire and Foster [43] designed a multidimensional poverty measurement method called the AF method. The method contains a deprivation dimension, weight distribution, multidimensional poverty identification, poverty aggregation and index decomposition, and is the basic method of MPI. This method has been the main tool used by domestic and foreign scholars to study multidimensional poverty thus far and has been recognized by many domestic and foreign scholars, organizations and governments [44]. This study uses the AF method to measure and decompose the health poverty of rural residents.

First, in the setting of the value of the health poverty dimension, let $M^{n,d}$ represent the n×d dimension matrix; *n* is the number of samples, and *d* is the dimension. Let $y\in M^{n,d}$ represent the different values of *n* individuals in the *d* dimensions, where $y_{ij}$ represents the value of the *i*-th rural resident in *j* dimension.

Second, define $Z_{j}$ as the deprivation value on the *j*-th dimension and the deprivation matrix as $g^{o}$ = $\left[g_{ij}^{0}\right]$, to represent the deprivation of the rural residents. If a rural resident is deprived under a certain indicator, the value of this indicator in the deprivation matrix is 1; otherwise the value is 0, indicating a non-deprivation status. The weight $w_{j}$ is set for each dimension, and $g_{ij}^{0}\cdot w_{j}$ represents the deprivation value of individual *i* in the *j* dimension.

Again, define a column vector to represent the sum of the deprivation values of rural resident *i* in all dimensions, i.e., the column vector $s_{i}$, $s_{i}=\sum_{j=1}^{d}W_{j}g_{ij}^{0}$. According to the critical value *K* of health poverty in the deprivation matrix, this study identified multidimensional health poverty for each sample: $s_{i}\left(k\right)=\{\begin{array}{cc} \sum_{j=1}^{d}W_{j}g_{ij}^{0}, & s_{i}\geq k\\ 0, & s_{i}<k \end{array}$. Additionally, $s_{i}\left(k\right)=\sum_{j=1}^{d}W_{j}g_{ij}^{0}$ indicates that individual *i* is poor under the critical value criterion *K*. We zeroed the deprivation value of non-healthy poor individuals to eliminate the interference of the deprivation information of the unhealthy poor individuals on the aggregation of health poverty, and called the deprivation matrix after zeroing as the censored matrix $g^{o}$ (*K*) (n×d).

The formula for calculating the HPI at this time is:

(1) $$M_{0}=HA_{1}=\frac{1}{n}\sum_{i=1}^{n}s_{i}\left(k\right)$$

(1) Equation: *H* is the adjusted incidence of multidimensional health poverty, *H* = qn, *q* is the number of identified multidimensional health poverty rural residents, and *n* is the total number; $A_{}$ is the average deprivation share, i.e., the intensity of deprivation, and $A_{}=\frac{1}{q}\sum_{i=1}^{n}s_{i}\left(k\right)$, i.e., the weighted average of the deprivation score values of the multidimensional health poverty population.

Finally, based on the $M_{0}$ calculated in Equation (1) above, the contribution of each indicator to health poverty is calculated:

(2) $$I_{j}=\frac{W_{i}\cdot CH_{i}}{M_{0}}$$

(2) Equation: $W_{i}$ is the weight value of the *i*-th column indicator, and $CH_{i}$ is the population rate of the *i*-th column indicator deprived in the deleted matrix.

### 3.2. MHPI Design

The traditional MPI includes 3 dimensions and 10 indicators [43], specifically: (1) health, including nutritional status and child mortality; (2) education, including children’s enrolment rates and educational attainment, and (3) living standard, including household electricity, sanitation, drinking water, flooring type cooking fuel, and asset ownership. The measurement dimension of health poverty should take greater account of the combined effects of economic, social, health environment, equity and efficiency and other factors [45]. Considering the current situation in China and the availability of data, this study expanded the MPI and designed an MHPI. The MHPI contains 5 dimensions, including (1) individual economic income, (2) individual health endowment, (3) individual health literacy and behavior, (4) healthy living environment, and (5) health service and security rights, as well as 17 secondary indicators. Table 1 details the dimensions, indicators, weights, and deprivation threshold.

The first index dimension is economic income. Select the per capita annual net income of households as the measurement index. Under the connotation of relative poverty, defining poverty according to a certain proportion of the average or median income of the society has become international common practice. This study considered those households whose per capita annual net income was lower than the median income of the sample group in the current year as experiencing economic poverty.

The second index dimension is individual health endowment. (1) The body mass index (BMI) is the most common tool used internationally to measure an individual’s weight to height ratio. This method was officially proposed by Keys et al. [46]. The calculation formula is as follows: BMI = weight (kg)/height^2^. On this basis, the critical value used to judge the degree to which Chinese adults are overweight and obese, as proposed by the Working Group on Obesity in China, is as follows: when the index is between 18.5 and 24, the adult is in a normal healthy state; outside this range is considered unhealthy. (2) Next, SRH is a variable frequently used in most literature to reflect the health status of individuals. In practice, SRH can comprehensively reflect the multidimensional nature and integrity of health [47,48]. In this study, those who rated themselves as unhealthy were considered to be in a state of health deprivation; the remaining respondents were considered to be in a state of health. (3) Chronic disease: disease is the biggest health threat faced by the rural poor in China [49]. The “Healthy China 2030 Blueprint” issued by the Chinese government in 2016 regarded major chronic diseases as indicators of premature mortality. Therefore, individuals with chronic diseases or those who were hospitalized due to illness in the previous 12 months were considered to be in a state of health deprivation in this study.

The third index dimension is individual health literacy and behavior. Referring to the indicators of the health literacy level of residents, developed by the Chinese Center for Health Education [50], and combined with the availability of data, this study designed six indicators representing the level of health literacy and behavior. Those indicators include: (1) whether an individual engages in smoking and alcohol abuse (smoking and drinking habits are generally considered unhealthy) [51]. (2) whether the person exercises. Regular physical activity contributes to good health [52]. (3) Health consumption expenditure: if the proportion of personal health consumption expenditure to total expenditure was lower than the median of the sample, these people were considered to be unwilling or unable to afford the normal level of health consumption expenditure [53], and their health literacy was low. (4) Choice of medical institution: those who can actively choose to go to formal medical institutions when seeking medical treatment were considered to have higher health literacy. Those who could not make such choices were considered to have lower health literacy and unscientific medical seeking behavior. (5) Hospitalization or not: those who were willing to accept hospitalization when they were sick were considered to have a high level of health literacy and scientific medical behavior, and vice versa. (6) Garbage dumping site selection: those who can take the initiative to choose a public garbage collection site to dump their garbage were considered to have high health literacy, while those who did not or could not make such choices were considered to have low health literacy and unscientific hygiene habits.

The fourth index dimension is the healthy living environment. Referring to the indicators of living environment in MPI, this study designed indicators in two dimensions: home environment and surrounding community environment. (1) Cooking water, cooking fuel and toilet type were selected as the proxy indicators of the home environment. The households whose cooking water was tap water and bottled water, the cooking fuel was non-firewood, and the toilet was flushing type were considered to have a healthy home environment. If the converse was true, the home environment was regarded as unhealthy [54,55]. (2) A community without highly-polluting enterprises within five kilometers of the residence was regarded as having a healthy community environment. Conversely, the community environment was regarded as unhealthy if highly-polluting enterprises were nearby [56]. 

The last index dimension is the health service and security rights. This type of indicator should include the two dimensions of residents’ health expenditure security and public health service sharing rights. The former mainly reflects the compensation of disease expenditure; the latter mainly reflects the degree of equalization of public health services and security. First, as a remedial measure to diversify the family’s economic risks [57], medical insurance can compensate for the economic losses caused by the risk of illness. This study uses medical insurance as a proxy indicator variable for health expenditure security, and considers rural residents who did not participate in any medical insurance schemes as being deprived of health expenditure security. Secondly, referring to the practice of Liu et al. [58], the number of medical and health workers per capita in the community, and the evaluation of the level of community medical services, are used as proxy indicator variables for the sharing rights of basic health services. The rural residents whose per capita number of medical and health workers was lower than the median and whose evaluation of the medical service level of the medical institutions was unsatisfactory were regarded as being deprived of the right to share health services.

Due to the large number of indicators involved in the study, the variance inflation factor (VIF) was used to test the multicollinearity of variables in the MHPI system. Table 2 reports the results. The VIF values range from 1.00 to 1.25, with an average VIF of 1.10; and all VIF values are below 5 [59]. These results indicate that there is no significant co-linearity in the variables.

### 3.3. Data Collection and Descriptive Analysis

This study uses data from China Family Panel Studies (CFPS), conducted by the Institute of Social Science Survey [60]. These data are from a large nationally representative micro-integrated family social tracking survey, which is conducted every two years and covers 25 provinces, municipalities and autonomous regions. The population of the sample area accounts for 94.5% of China’s total population, and the target sample size is 16,000 households. Hence, this study has the advantages of large sample size and wide coverage. The survey adopts a stratified multi-stage sampling design (sampling protocol is publicly available at www.isss.pku.edu.cn/cfps/ (accessed on 13 September 2022)) and contains four sections: community questionnaire, family questionnaire, adult questionnaire and children’s questionnaire. The survey’s design and various sections fully reflect the changes in China’s economy, society, population, education development and personal health [61]. This survey has been ethically reviewed (approval number: IRB00001052-14010), thereby providing real and reliable data for academic research and public policy analysis in various fields of the social sciences [62].

**Figure 1:**
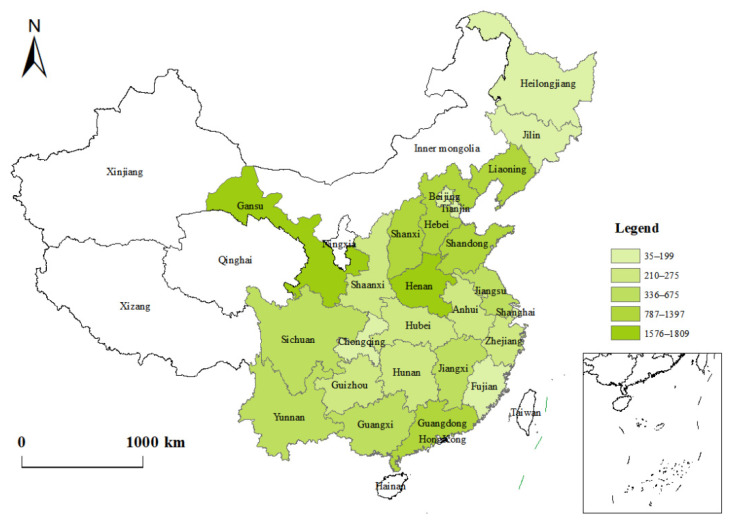
Sample distribution.

Since the 2010 baseline survey, CFPS has conducted five rounds of follow-up surveys. This study uses data collected by CFPS in 2016 and 2018. Indicators such as “SRH”, “chronic diseases”, “smoking” and “drinking” come from the adult questionnaire. Indicators such as “water for cooking”, “fuel for cooking” and “toilet type” come from the family questionnaire. Indicators such as “highly polluting enterprises” and “number of medical and health workers” come from the community questionnaires. Each part of the questionnaires contains a wide range of questions, which fully reflects the various aspects of rural residents’ personal health and family life. As such, the questionnaires can better measure the diversity of rural residents’ health poverty in different regions of China. In order to improve the accuracy and integrity of the characteristic variables and data after questionnaire merging, this study conducted descriptive statistics on the basic characteristics of the samples; any samples with missing data and obvious outliers were eliminated. Ultimately, a total of 13,151 samples from 25 provinces were obtained, and the specific sample distribution is shown in Figure 1. Detailed socio-demographic statistics are shown in Table 3.

**Figure 2:**
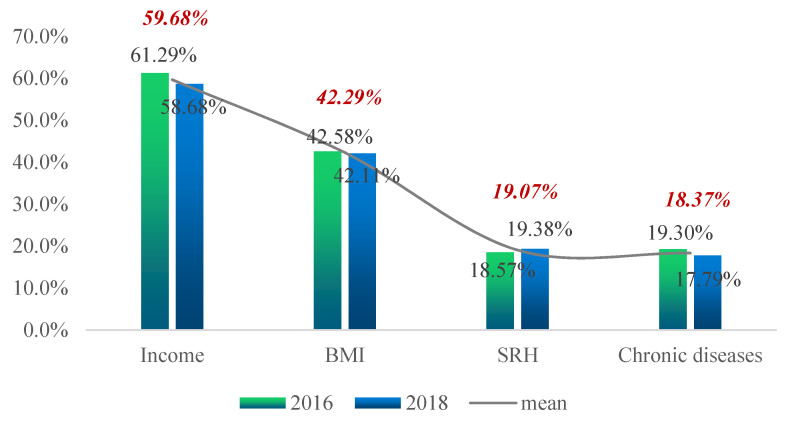
Proportion of the poor for each indicator of the economic income dimension and the individual health endowment dimension.

**Figure 3:**
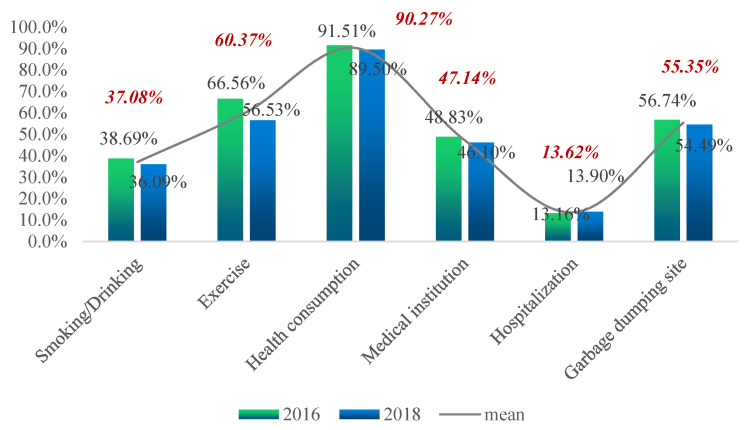
Proportion of the poor for each indicator of the individual health literacy and behavioral dimension.

**Figure 4:**
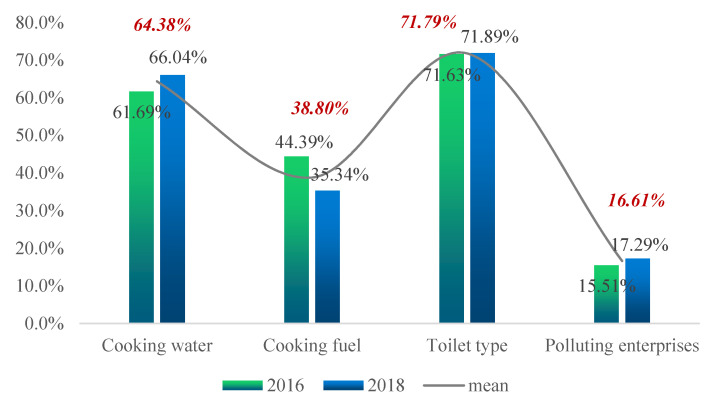
Proportion of the poor for each indicator of the healthy living environment dimension.

**Figure 5:**
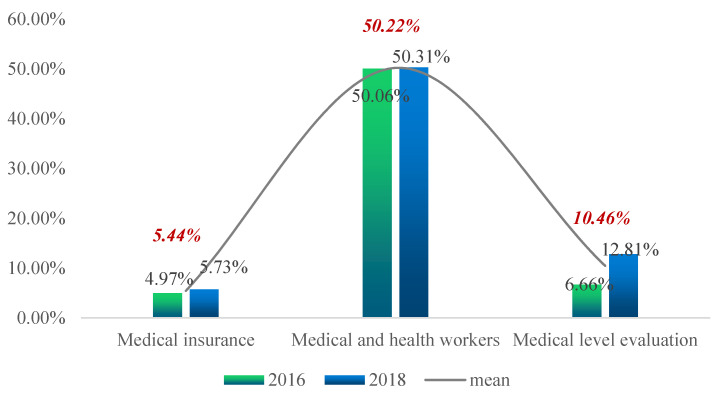
Proportion of the poor for each indicator of the health service and security rights dimension.

Figure 2, Figure 3, Figure 4 and Figure 5 show the proportion of the population in deprivation (i.e., health poverty) in the total sample under each of the five dimensions; the data for two years are also compared.

Figure 2 shows that, from the two-year mean, the proportion of the population that had “per capita annual net income of households” below the relative poverty line was the highest, at 59.86%. The proportion of the population suffering from “chronic diseases” was the lowest, at 18.37%. By comparing the two-year data, one can see that, except for the increase in the proportion of health poverty people in “SRH”, all other indicators showed a downward trend.

Figure 3 shows that, from the two-year mean, the proportion of health poverty people in the indicator of “health consumption” was the highest, at 90.27%. The proportion of health poverty people in the indicator of “hospitalization or not” was the lowest, at 13.62%. The other indicators were roughly the same. By comparing the two-year data, one can see that, except for the slight increase in the proportion of the health poverty population in the indicator of “hospitalization or not”, the proportions of the other indicators decreased significantly.

Figure 4 shows that, from the two-year mean, the proportion of health poverty people in the indicator of “toilet type” was the highest, at 71.79%. The proportion of “high-pollution enterprises” was the lowest, at 16.61%. By comparing the two-year data, one can see that the proportion of health poverty people in the indicator of “cooking water” and “highly-polluting enterprises” increased slightly, while the rest decreased.

Figure 5 shows that, from the two-year mean, the proportion of health poverty people in the indicator of “number of medical and health workers” was the highest, at 50.22%. The proportion of those in “medical insurance” was the lowest, at 5.44%. By comparing the two-year data, one can see that the proportions of the health poverty in the three indicators increased to different degrees. The highest increase was the “medical level evaluation”, with an increase of 6.15 percentage points.

## 4. Results

### 4.1. Calculation of Health Poverty

#### 4.1.1. Overall Situation

This study uses the AF method to measure the health poverty deprivation of rural residents in China, in 2016 and 2018. A horizontal comparison to observe the changes is also made. Table 4 shows that, as the *K* value increased, the MHPI (*M*_0_) gradually decreased; the incidence of health poverty (*H*) also showed a downward trend. However, the deprivation intensity (*A*) showed an upward trend. According to the United Nations’ ‘*K* value use standard’, *K* ≥ 0.3 is often used in the multidimensional poverty measurement. That is, individuals whose deprivation index is equal to or more than 30% are identified as being in multidimensional health poverty. Specifically, in 2016, when *K* = 0.3, the *M*_0_ of rural residents was 0.363. That is to say, more than 36.3% of rural households had a health indicator deprivation of at least 30% of and *H* was 75.03%. The *A* (i.e., the weighted average of the deprivation indicator score for health poverty) was 48.39%. In 2018, the *M*_0_ was 0.353. That is, more than 35.3% of rural residents had at least 30% deprivation of health indicators. At this time, *H* was 73.78% and *A* was 47.82%, both showing a downward trend (compared with 2016). In 2016, when *K* = 0.6, the *M*_0_ of rural residents was 0.066, *H* was 10.08%, and *A* was 65.27%. By 2018, these three indicators had decreased to 0.064, 9.81%, and 64.77%, respectively.

It is important to note that the second half of Table 4 shows the 95% confidence intervals, standard errors, and differences in means for *M*, *H*, and *A* in 2016 and 2018. The confidence intervals and standard errors provide the possibility to measure the accuracy of the interval estimates. At the same time, the comparison of the means between the two years revealed that *M*, *H* and *A* were all reduced.

#### 4.1.2. Heterogeneity Analysis

To measure whether significant differences exist between groups with different demographic characteristics, this study conducted a heterogeneity analysis on the MHPI, with *K* = 0.3 as an example. The results are shown in Table 5.

In terms of age group, *M*_0_, *H*, and *A* all increased with age, especially in the elderly group (over 61 years old). Taking the 2018 data as an example, the *H* of the elderly group reached 83.57%, or 20.32 percentage points higher than the *H* of the young group (under 40 years old). By comparing the two-year data, one can see that only the *M*_0_ and *H* of the middle-aged group decreased at the same time, and the health poverty situation improved. Other groups experienced increases to varying degrees; the deterioration of health poverty in the young group deserves special attention.

In terms of household size group, the *M*_0_, *H*, and *A* of medium-sized households were all lower. The health poverty status of households with six or more people was also the worst, with *H* reaching 77.92% in 2018. By comparing the two-year data, one can see that all indices decreased to varying degrees, and the health poverty situation has improved significantly.

In terms of gender group, the *M*_0_, *H*, and *A* of females were all better than those of males. However, by comparing the two-year data, one can see that the improvement in health poverty of males was greater than that of females. In terms of education group, the higher the education level, the better the health poverty status. In terms of marital status group, the health poverty status of rural residents in the married state was slightly better than the status of those in the unmarried state, but the difference was not significant.

### 4.2. Decomposition

#### 4.2.1. Decomposition by Region

**Figure 6:**
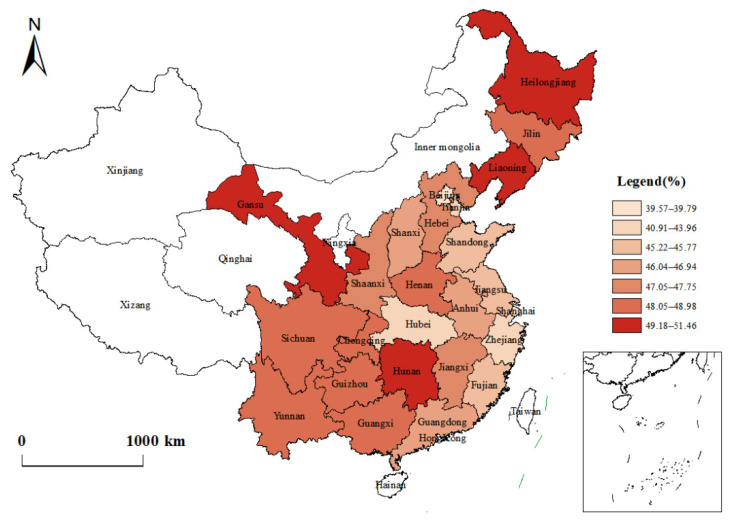
Regional decomposition of health poverty deprivation intensity.

In order to better reveal the relationship between the health poverty status of rural residents and the level of local economic development, this study still takes *K* = 0.3 as an example, and decomposes the HPI by region and province. The results are shown in Table 6 and Figure 6.

First, from the distribution of the Eastern, Central and Western regions, the indicators (such as *H* and *A*) of rural residents in the Eastern region were the lowest, followed by the Central region. The highest indicators were in the Western region. Judging from the changes in the two-year data, various indices of rural residents in the Eastern and Western regions declined to varying degrees, but the decline rate of health poverty in the Central region increased slightly, at −0.017%.

Second, from the perspective of different provinces, in 2018, the province with the highest *H* and the deepest deprivation intensity of rural residents in the Eastern region was Liaoning, at 49.04% and 80.42%, respectively. These rates were higher than those of Guangxi, which were 47.36% and 78.74%, respectively. However, according to the national provincial ranking of rural residents’ disposable income, Liaoning ranked 10th, while Guangxi ranked 20th. In the Central region, the province with the lowest *H* and lighter deprivation intensity was Hubei, followed by Shanxi. However, Shanxi’s rank for the disposable income of rural residents was only 21st in the country, and last in the Central region. The *H* of the four provinces of Henan, Heilongjiang, Hunan and Jilin, all in the Central region, increased slightly. Notably, the *H* of Heilongjiang was as high as 52.14%, the highest in the country. In the Western region, the *H* increased slightly in Guizhou and Yunnan, and decreased in other provinces. The lowest *H* in Shaanxi was 47.7%, which was even lower than in Chongqing and Sichuan, which ranked ahead of rural residents’ disposable income. The *H* in Gansu was the highest, at 50.27%.

#### 4.2.2. Decomposition by Indicators

Table 7 reports the contribution rate of each dimensional indicator to health poverty at *K* = 0.3. According to the mean data, the largest contribution rate was still the economic income dimension, and the contribution rate of the “per capita annual net income of households” indicator was 33.3%. The second largest rate was that of the healthy living environment dimension, which includes “water for cooking” (6.56%), “fuel for cooking” (4.9%) and “toilet type” (8.14%). This was followed by the individual health literacy and behavior dimension, including indicators such as “health consumption expenditure” (6.43%), “whether to exercise” (4.46%), “garbage dumping site selection” (4.25%), and “choice of medical institution” (3.43%). The contribution rate of the “number of medical and health workers” in the dimension of health service and security rights was also as high as 7.42%.

By comparing the two-year data, one can see that the indicators with increased contribution rates include “BMI” and “SRH” in the dimension of individual health endowment, “hospitalization or not” in the dimension of individual health literacy and behavior, and “water for cooking”, “toilet type” and “highly-polluting enterprises” in the dimension of healthy living environment. Furthermore, the contribution rates of all indicators in the dimension of health service and security rights increased over these two years. However, in the mean comparison, the contribution rates of indicators with higher contribution rates, such as the “per capita annual net income of households” and “health consumption expenditures” all showed a downward trend.

## 5. Discussion

### 5.1. Research Findings

This study uses the MHPI to measure and decompose the health deprivation status and changes of rural residents in China. In terms of the overall situation, the health poverty status of rural residents improved significantly from 2016 to 2018, with indicators such as *M*_0_, *H* and *A* showing a year-on-year decline. These findings indicate that the effect of rural health poverty alleviation measures in China has been very significant in recent years; the well-being of rural residents’ health has been greatly improved. During the study period, health poverty status was highly correlated with the age and education level of rural residents. The older the age and the lower the education level of the residents was, the higher was the incidence of poverty. The health poverty status of medium-sized households was the best, while the health poverty status of the rural female group was slightly better than that of the male group. The health poverty status had little relationship with whether the rural residents were married. These findings are consistent with most of the existing literature measuring the extent and improvement of multidimensional poverty in China [63,64,65].

In terms of regional decomposition, the rural areas in the East had the lowest health poverty severity, followed by the Central region. The highest degree of severity was found in the Western region. This finding shows that the rural areas with higher economic development levels have a higher degree of equalization of public services in terms of income, home and community health environment, medical and health security, etc. [66,67]. However, very few studies have focused on the health literacy and behaviors of poor rural residents. This study found that the more economically developed the area, the higher the health literacy of rural residents and the more scientific the health behaviors [68]. By comparing the two-year data, one can see that, during the study period, the various health poverty indices in the Eastern and Western regions decreased to varying degrees, but the incidence of health poverty in the Central region increased slightly. These findings indicate that the regional distribution of health disparities has undergone new changes. From the provincial decomposition, the degree of health poverty can be seen to no longer be closely related to the level of local economic development. Some provinces, such as Guangxi, Hubei and Shaanxi, improved their health poverty situations faster than the local economic development. In other provinces, such as Heilongjiang, Liaoning and Henan, the rate of improvement of health poverty was not satisfactory. These findings indicate that, in addition to the factors of regional economic development, the health poverty status is also closely related to the local social and cultural environment, and, to a considerable extent, to the government’s poverty governance capacity.

In terms of the decomposition of indicators, the contribution rate of “per capita annual net income of households” in the economic income dimension was the largest [69]. The contribution rates of “water for cooking”, “fuel for cooking” and “toilet type” in the healthy living environment dimension were relatively large [70]; the contribution rates of “health consumption expenditure”, “whether to exercise” and “garbage dump site selection” in the individual health literacy and behavior dimension were relatively large. By comparing the two-year data, one can see that the contribution rate of the “per capita annual net income of households” decreased during the study period, while the contribution rates of all indicators in the dimension of health service and security rights increased. The contribution rates of most indicators in other dimensions also increased to varying degrees. These findings indicate that, although the marginal contribution of economic poverty alleviation is gradually decreasing [71,72], health consumption expenditure is still constrained by the economic income of rural residents. The equalization of health services and security has shifted to a policy focus, and community environmental management has also become an important aspect of health poverty governance [13,73]. Individual health literacy and behavior gradually played an important endogenous role in poverty alleviation during the study period, and more and more literature has begun to focus on this point [74,75].

### 5.2. Policy Implications

Based on the above findings, the following specific policy recommendations are proposed:

Firstly, the MHPI constructed in this study accurately and comprehensively measures the health deprivation status of the rural poor in China, from both the supply and demand levels. The comparative analysis and heterogeneity analysis of the two periods of data show that the health poverty situation of the rural population in China has been greatly improved. However, there were still differences in the situation that related to gender, age and household size. During the two sampled years, the contribution rate of health poverty indicators varied greatly. The marginal contribution of rural residents’ individual health literacy and behavior, living environment, and equalization of health services and security also increased. Therefore, it is recommended to construct a multi-dimensional rural health poverty identification index system that covers physical, mental, behavioral, living environment and public health service security. On this basis, a health poverty early warning mechanism should be established in a timely manner. The goal should be to dynamically monitor and assist the health deprivation status of the rural poor, and eliminate the risk of poverty and any return to poverty.

Secondly, through the analysis of index decomposition, the largest contributor to health poverty was still economic income. Recent evidence suggests that many countries have vigorously developed supplementary private pensions. However, these measures cannot raise private saving so that poverty in old age decreases [76,77]. Therefore, non-contributory pensions become an optional path. It can not only reduce economic poverty level but also improve the health [78,79,80]. In addition, individual health literacy and behavior were found to play an important role in endogenous poverty alleviation. Therefore, this study recommends carrying out publicity and education related to health knowledge through new social media. The objective should be to scientifically guide people’s healthy lifestyles, regulate health behaviors, and improve health literacy.

Finally, indicators such as the equalization of health services and security, community environmental governance and others have a large contribution rate. However, this also means that these indicators pose a significant risk of both poverty and a return to poverty. As such, these indicators should become the policy focus of future health poverty governance. Therefore, grass-roots governments should accelerate the improvement of rural habitats, attach importance to humanistic care, improve the living environment of the rural community, enhance the ability of grass-roots village governance, and build beautiful and livable villages. China’s central government should provide high-quality medical service resources to remote and poor areas, promote the equalization of health services and security, and reduce regional disparities in health poverty. Particular attention should be paid to those areas where the improvement of health poverty is slow, and efforts should be made to prevent the emergence and spread of secondary poverty.

## 6. Conclusions and Limitations

In the era of multidimensional poverty, health is an important dimension for effective poverty alleviation in most countries around the world. From the perspective of the supply side and demand side, this study constructs an MHPI and uses CFPS to measure and decompose the health poverty of rural residents in China. The study finds that, after years of health poverty alleviation projects, the health and poverty status of Chinese rural residents has been greatly improved. However, significant regional disparities still exist in the extent of health poverty. The rate of improvement in health poverty in the rural areas of some provinces lags behind the level of local economic development. This clearly indicates that health poverty is also closely related to the local social and cultural environment and the government’s governance capacity. The marginal contribution of economic poverty alleviation is diminishing. The equalization of health services and security has shifted to a policy focus. Community environmental management has also become an important aspect of health poverty governance, and individual health literacy and behavior have played and will continue to play an important role in endogenous poverty alleviation. 

It should be acknowledged that there is still room for improvement in this study. First, based on the limited availability of data, this study used only two years of data to measure rural health poverty in China. Considering that the measurement results in different years may be different, more data from different years are needed to better reveal the changes of health poverty and provide a more scientific data basis for policymakers. Second, although this study measures and decomposes the health literacy and behavior of poor individuals as important indicators, the causal relationship between these indicators and the occurrence of health poverty was not examined. Future research may attempt to reveal the internal logic between these indicators and health poverty.

## Figures and Tables

**Table 1:** Multidimensional health poverty index (MHPI).

Dimension	Indicators	Weights	Deprivation Threshold
Economic income	Per capita annual net income of households	1/5	Below the annual poverty line = 1; otherwise = 0
Individual health endowment	BMI	1/15	Not in the interval of (18.5–24) = 1; otherwise = 0
	SRH	1/15	Self-rated as unhealthy = 1; otherwise = 0
	Chronic diseases	1/15	With chronic disease = 1; otherwise = 0
Individual health literacy and behavior	Smoking and alcohol abuse	1/30	Either smoking or drinking = 1; otherwise = 0
	Exercise	1/30	No exercise in one week = 1; otherwise = 0
	Health consumption expenditure	1/30	The proportion of personal medical care expenditure to total expenditure is lower than the median of the total sample = 1; otherwise = 0
	Choice of medical institution	1/30	Select community health service station, village health office, clinic = 1; otherwise = 0
	Hospitalization or not	1/30	No hospitalization if sick = 1; otherwise = 0
	Garbage dumping site selection	1/30	Select nearby river ditches, around houses, soil pits, and dump everywhere = 1; otherwise = 0
Healthy living environment	Water for cooking	1/20	Use river and lake water, well water, rainwater, cellar water, pond water/mountain spring water = 1; otherwise = 0
	Fuel for cooking	1/20	Use firewood, coal = 1; otherwise = 0
	Toilet type	1/20	Indoor non-flushing toilet, outdoor non-flushing toilet, non-flushing public toilet = 1; otherwise = 0
	Highly-polluting enterprises	1/20	Highly-polluting enterprises within five kilometers of residence = 1; otherwise = 0
Health service and security rights	Medical insurance	1/15	Not enrolled in any medical insurance = 1; otherwise = 0
	Number of medical and health workers	1/15	The number of medical and health workers per capita in the community is lower than the median of the total sample = 1; otherwise = 0
	Medical level evaluation	1/15	Dissatisfied with the medical level of local medical institutions = 1; otherwise = 0

**Table 2:** Collinearity assessment results.

Indicators	VIF	1/VIF
Garbage dumping site selection	1.25	0.802
Toilet type	1.24	0.808
SRH	1.23	0.816
Fuel for cooking	1.21	0.83
Chronic diseases	1.19	0.841
Hospitalization or not	1.15	0.87
Per capita annual net income of households	1.08	0.925
Water for cooking	1.07	0.932
Highly-polluting enterprises	1.06	0.947
Choice of medical institution	1.05	0.952
Number of medical and health workers	1.04	0.964
Health consumption expenditure	1.03	0.971
Smoking and alcohol abuse	1.02	0.979
Exercise	1.02	0.98
BMI	1.01	0.989
Medical level evaluation	1.01	0.992
Medical insurance	1.00	0.997
Mean VIF	1.1	

**Table 3:** Socio-demographic statistics.

Characteristic	Demographic	Frequency	%
Age (years)	Young group: under 40	2676	20.35
	Middle-aged group: 41–60	6038	45.91
	old group: 61 and above	4437	33.74
Household size	3 or fewer	5542	42.14
	4–5	4453	33.86
	6 and more	3156	24.00
Gender	Male	6589	50.10
	Female	6562	49.90
Education	Illiterate/semi illiterate	4962	37.73
	Primary school	3904	29.69
	Junior high school	3157	24.01
	Senior high school and above	1128	8.58
Marital status	Married	11,230	85.39
	Unmarried	1921	14.61

Note: The data come from CFPS in 2016 and 2018.

**Table 4:** Multidimensional health poverty index under different thresholds.

*K* Value	Multidimensional Health Poverty Index (*M*_0_)		Incidence of Health Poverty (*H*, *%*)		Deprivation Intensity (*A, %*)	
	2016	2018	2016	2018	2016	2018
*K* = 0.1	0.419	0.410	99.32	99.21	42.15	41.29
*K* = 0.2	0.407	0.396	92.43	90.89	44.06	43.57
*K* = 0.3	0.363	0.353	75.03	73.78	48.39	47.82
*K* = 0.4	0.300	0.286	57.34	54.72	52.39	52.22
*K* = 0.5	0.182	0.174	31.33	30.03	57.95	57.83
*K* = 0.6	0.066	0.064	10.08	9.81	65.27	64.77
*K* = 0.7	0.012	0.011	1.67	1.44	73.31	73.39
[95% Conf. Interval]	[0.097, 0.403]	[0.093, 0.391]	[16.343, 88.571]	[15.514, 87.309]	[44.214, 65.363]	[43.62, 65.206]
Std. Err.	0.062	0.061	14.759	14.671	4.322	4.411
MeanDiff	−0.008		−1.046		−0.376	

Note: The data come from CFPS in 2016 and 2018.

**Table 5:** Heterogeneity analysis of multidimensional health poverty index at *K* = 0.3.

	Multidimensional Health Poverty Index (*M*_0_*)*		Incidence of Health Poverty (*H*, *%*)		Deprivation Intensity (*A*, *%*)	
	2016	2018	2016	2018	2016	2018
Age (years)						
Young group	0.261	0.285	58.39	63.25	44.62	44.98
Middle-aged group	0.352	0.343	74.20	72.38	47.39	47.42
Elderly group	0.419	0.417	82.81	83.57	50.65	49.89
Household size						
3 or fewer	0.352	0.348	72.37	72.71	48.63	47.83
4–5	0.367	0.343	76.31	71.87	48.05	47.79
6 and more	0.380	0.373	78.38	77.92	48.49	47.83
Gender						
Male	0.375	0.363	76.82	75.29	48.78	48.25
Female	0.350	0.343	72.97	72.39	47.92	47.40
Education						
Illiterate/semi illiterate	0.400	0.431	81.08	85.89	49.36	50.20
Primary school	0.323	0.358	68.81	74.76	46.87	47.94
Junior high school	0.291	0.319	62.39	68.65	46.61	46.44
Senior high school and above	0.192	0.278	45.95	61.65	41.86	45.13
Marital status						
Married	0.360	0.353	74.76	73.85	48.17	47.83
Unmarried	0.382	0.351	76.73	73.43	49.73	47.74

Note: The data come from CFPS in 2016 and 2018.

**Table 6:** Regional decomposition of the incidence of health poverty.

Region	Province	Per capita Disposable Income of Rural Residents (Yuan)		Incidence of Health Poverty (*H*, *%*)		Health Poverty Decline Rate (*%*)
		2016	2018	2016	2018	-
Eastern	Beijing	22,309.5	26,490.3	38.61	40.50	−0.049
	Fujian	14,999.2	17,821.2	45.60	44.92	0.015
	Guangdong	14,512.2	17,167.7	46.43	45.75	0.015
	Guangxi	10,359.5	12,434.8	49.24	47.36	0.038
	Jiangsu	17,605.6	20,845.1	48.01	44.10	0.081
	Hebei	11,919.4	14,030.9	47.79	47.28	0.011
	Liaoning	12,880.7	14,656.3	49.40	49.04	0.007
	Shanghai	25,520.4	30,374.7	41.58	38.15	0.082
	Shandong	13,954.1	16,297.0	46.37	45.15	0.026
	Zhejiang	22,866.1	27,302.4	41.71	40.46	0.030
	Tianjin	20,075.6	23,065.2	45.19	42.80	0.053
	Mean	17,000.2	20,044.1	45.45	44.14	0.029
Central	Anhui	11,720.5	13,996.0	48.13	46.19	0.040
	Henan	11,696.7	13,830.7	48.77	49.08	−0.006
	Heilongjiang	11,831.9	13,803.7	49.92	52.14	−0.044
	Hubei	12,725.0	14,977.8	44.07	43.90	0.004
	Hunan	11,930.4	14,092.5	49.28	49.81	−0.011
	Jilin	12,122.9	13,748.2	48.04	49.54	−0.031
	Jiangxi	12,137.7	14,459.9	48.07	46.33	0.036
	Shanxi	10,082.5	11,750.0	48.02	46.08	0.040
	Mean	11,781.0	13,832.4	48.04	48.88	−0.017
Western	Gansu	7456.9	8804.1	51.00	50.27	0.014
	Guizhou	8090.3	9716.1	48.28	49.20	−0.019
	Shaanxi	9396.4	11,212.8	47.82	47.70	0.003
	Sichuan	11,203.1	13,331.4	49.02	47.76	0.026
	Yunnan	9019.8	10,767.9	47.24	48.46	−0.026
	Chongqing	11,548.8	13,781.2	48.62	48.44	0.004
	Mean	9452.6	11,268.9	48.66	48.64	0.000

Note: The data come from CFPS in 2016 and 2018, and the China Statistical Yearbook (electronic edition) in 2017 and 2019, http://www.stats.gov.cn/tjsj/ndsj/2017/indexch.htm (accessed on 8 September 2022), and http://www.stats.gov.cn/tjsj/ndsj/2019/indexch.htm (accessed on 8 September 2022).

**Table 7:** Decomposition of the contribution rate of each indicator at *K* = 0.3 (%).

Dimension	Indicators	2016	2018	Mean
Economic income	Per capita annual net income of households	33.50	33.10	33.30
Individual health endowment	BMI	6.40	6.55	6.48
	SRH	3.27	3.44	3.36
	Chronic diseases	3.22	3.12	3.17
Individual health literacy and behavior	Whether engaged in smoking and alcohol abuse	2.79	2.64	2.72
	Whether exercising or not	4.78	4.13	4.46
	Health consumption expenditure	6.45	6.40	6.43
	Choice of medical institution	3.53	3.32	3.43
	Hospitalization or not	1.08	1.15	1.12
	Garbage dumping site selection	4.30	4.20	4.25
Healthy living environment	Water for cooking	6.27	6.84	6.56
	Fuel for cooking	5.38	4.41	4.90
	Toilet type	8.06	8.21	8.14
	Highly-polluting enterprises	1.69	1.89	1.79
Health service and security rights	Medical insurance	0.80	0.95	0.88
	Number of medical and health workers	7.32	7.52	7.42
	Medical level evaluation	1.14	2.14	1.64

Note: The data come from CFPS in 2016 and 2018.

## Data Availability

The data analyzed in this study are openly accessible on the website of the ISSS at Peking University (http://www.isss.pku.edu.cn/cfps/ (accessed on 13 September 2022)).
